# Supplementary material for: Comparative genomics provides new insights into the diversity, physiology, and sexuality of the only industrially exploited tremellomycete: Phaffia rhodozyma
Source: BMC Genomics. 2016 Nov 9;17:901. doi: 10.1186/s12864-016-3244-7 (PMC5103461; doi:10.1186/s12864-016-3244-7)
Supplement: Additional file 6: — List of orphan genes with links to PFAM (related to Additional file 1: Table S1). (ZIP 1428 kb) [file 12864_2016_3244_MOESM6_ESM.zip › BLAST_HTML_FTR/G03849_P.html]

BLAST Search Results


```
BLASTP 2.2.27+


Reference:
Stephen F. Altschul, Thomas L. Madden, Alejandro A. Schäffer,
Jinghui Zhang, Zheng Zhang, Webb Miller, and David J. Lipman (1997),
"Gapped BLAST and PSI-BLAST: a new generation of protein database
search programs", Nucleic Acids Res. 25:3389-3402.


Reference for
composition-based statistics:
Alejandro A. Schäffer, L. Aravind, Thomas L. Madden, Sergei
Shavirin, John L. Spouge, Yuri I. Wolf, Eugene V. Koonin, and
Stephen F. Altschul (2001), "Improving the accuracy of PSI-BLAST
protein database searches with composition-based statistics and
other refinements", Nucleic Acids Res. 29:2994-3005.


Database: nr
           71,551,133 sequences; 26,053,659,533 total letters


Query= G03849_P

Length=336
                                                                      Score     E
Sequences producing significant alignments:                          (Bits)  Value

emb|CDZ96324.1|  hypothetical protein [Xanthophyllomyces dendrorh...   654    0.0  
emb|CDZ96585.1|  hypothetical protein [Xanthophyllomyces dendrorh...  76.6    4e-12
emb|CCU97607.1|  unnamed protein product [Malassezia sympodialis ...  39.7    5.7  


 >emb|CDZ96324.1| hypothetical protein [Xanthophyllomyces dendrorhous]
Length=332

 Score =  654 bits (1687),  Expect = 0.0, Method: Compositional matrix adjust.
 Identities = 332/332 (100%), Positives = 332/332 (100%), Gaps = 0/332 (0%)

Query  4    MAAHPSLPPTPSNAGKHLFSTRPRQQPPRARSASASPRPKQSPAIGPSFPCSPLAKNSQS  63
            MAAHPSLPPTPSNAGKHLFSTRPRQQPPRARSASASPRPKQSPAIGPSFPCSPLAKNSQS
Sbjct  1    MAAHPSLPPTPSNAGKHLFSTRPRQQPPRARSASASPRPKQSPAIGPSFPCSPLAKNSQS  60

Query  64   RLSQSRPVSPVKQTSELTLWPPTTAPNADERRSRSRGAKKGARGKEKKIVLEEAFSSLEL  123
            RLSQSRPVSPVKQTSELTLWPPTTAPNADERRSRSRGAKKGARGKEKKIVLEEAFSSLEL
Sbjct  61   RLSQSRPVSPVKQTSELTLWPPTTAPNADERRSRSRGAKKGARGKEKKIVLEEAFSSLEL  120

Query  124  DAGVPSEADVWDMPIKASGRDVLTWQQTSSSSSPSKLPSSPRPRAARSQQGQPVSTSKSI  183
            DAGVPSEADVWDMPIKASGRDVLTWQQTSSSSSPSKLPSSPRPRAARSQQGQPVSTSKSI
Sbjct  121  DAGVPSEADVWDMPIKASGRDVLTWQQTSSSSSPSKLPSSPRPRAARSQQGQPVSTSKSI  180

Query  184  APFSRSSNQPLAGPFHHASSSSITAVSSNSNLTWQQALFETAHPASSRPKIRRRASLGNE  243
            APFSRSSNQPLAGPFHHASSSSITAVSSNSNLTWQQALFETAHPASSRPKIRRRASLGNE
Sbjct  181  APFSRSSNQPLAGPFHHASSSSITAVSSNSNLTWQQALFETAHPASSRPKIRRRASLGNE  240

Query  244  SGTAPVHLPRLGVPVKSNSIHHSSSMKNKRVSGSPPNNGERVNGSSYPAGTVGSDSGGVR  303
            SGTAPVHLPRLGVPVKSNSIHHSSSMKNKRVSGSPPNNGERVNGSSYPAGTVGSDSGGVR
Sbjct  241  SGTAPVHLPRLGVPVKSNSIHHSSSMKNKRVSGSPPNNGERVNGSSYPAGTVGSDSGGVR  300

Query  304  RPEGEINTYAGGTFQNAPLAGDLPKPRFGARP  335
            RPEGEINTYAGGTFQNAPLAGDLPKPRFGARP
Sbjct  301  RPEGEINTYAGGTFQNAPLAGDLPKPRFGARP  332


>emb|CDZ96585.1| hypothetical protein [Xanthophyllomyces dendrorhous]
Length=426

 Score = 76.6 bits (187),  Expect = 4e-12, Method: Compositional matrix adjust.
 Identities = 73/222 (33%), Positives = 102/222 (46%), Gaps = 55/222 (25%)

Query  136  MPIKASGRDVLTWQQTSSSSSPSK-LPSSPRPRAARSQQGQPVSTSKSIAPFSRSSNQPL  194
            MP++  G DVLTWQQTS  S+P+K  P  P PRA  ++       ++SI+P         
Sbjct  236  MPLEKRGTDVLTWQQTSPPSAPTKPTPKMPSPRAQVNRL-----QTRSISP---------  281

Query  195  AGPFHHASSSSITAVSSNSNLTWQQALFETAHPASSRPKIRRRASL--------------  240
                  + + SI+   S +NLTWQQALFE++ P     K    A+L              
Sbjct  282  ------SPAVSISTPPSATNLTWQQALFESSAPVVVSKKKNPTANLQRRSSFESSLSSSA  335

Query  241  ------GNESGTAPVHLPRLGVPVKSNSI--HHSSSMKNKRVSGSPPNNGERVNGSSYPA  292
                  G  S TA         P K N+    H  +  N+R S     + + ++ S+   
Sbjct  336  PPAQFAGPTSSTA-------TRPAKGNTFPRAHHLTQNNQRASS---GHAKSISLSTLGL  385

Query  293  GTVGSDSGGVRRPEGEINTYAGGTFQNAPLAGDLPKPRFGAR  334
                 +SG     +G +  YAG  FQN+PLAGDLPKPRFG +
Sbjct  386  AGGSIESGADNSAKGPL--YAGSVFQNSPLAGDLPKPRFGLK  425


>emb|CCU97607.1| unnamed protein product [Malassezia sympodialis ATCC 42132]
Length=1170

 Score = 39.7 bits (91),  Expect = 5.7, Method: Compositional matrix adjust.
 Identities = 71/274 (26%), Positives = 103/274 (38%), Gaps = 78/274 (28%)

Query  77   TSELTLWPPTTA----------PNADERRSRSRGAKKGARGKEKKIVLEEAFSSLELDAG  126
            T  L L+P T+           PN      RS+  ++ A       +L+  +  +  D G
Sbjct  90   TRVLALYPDTSCFYWATVQGGGPNTQAHTLRSKSQRRDAD------MLQAPYKLMFYDDG  143

Query  127  VPSEADVWDMPIKASGRDVLTWQQTSSSSSPSKLPSSPRPRAARSQQGQPVSTSKSIAPF  186
                ADV  +P       V+TWQQ   + S ++   +PR +  ++ +  P    K+ AP 
Sbjct  144  ----ADVKQVPAYL----VMTWQQQMMTPSWNQ---APRKKKEKNTEA-PKKKEKTKAP-  190

Query  187  SRSSNQPLAGPFHHASSSSITAVSSNSNLTWQQALF---ETAHPASSRP-KIRRRASLGN  242
                              ++  V   SN+TWQQ LF   +   PA       R   + G 
Sbjct  191  ------------------AVEKV--GSNMTWQQELFLQSKRVGPAFDHAADARDEQTFGG  230

Query  243  ESGTAPVHLP--RLGVPVKSNSIHHSSSMKNKRVSGSPPNNGERVNGSSYPAGTVGSDSG  300
            ES   P   P    G P +  +     S KN RV    PN  ++  GS+  A        
Sbjct  231  ESALRPKSAPPGTTGTPERRKAGKSKGSNKNTRV----PNTPQKKQGSAPVA--------  278

Query  301  GVRRPEGEINTYAGGTFQNAPLAGDLPKPRFGAR  334
                       YAG TF N+P A  LP P+F  R
Sbjct  279  -----------YAGPTFHNSPSAASLPTPKFANR  301


Lambda      K        H        a         alpha
   0.309    0.124    0.364    0.792     4.96 

Gapped
Lambda      K        H        a         alpha    sigma
   0.267   0.0410    0.140     1.90     42.6     43.6 

Effective search space used: 2821146113250


  Database: nr
    Posted date:  Sep 23, 2015 12:05 AM
  Number of letters in database: 26,053,659,533
  Number of sequences in database:  71,551,133


Matrix: BLOSUM62
Gap Penalties: Existence: 11, Extension: 1
Neighboring words threshold: 11
Window for multiple hits: 40
```
